# Modified turn-up technique for proximal anastomosis in acute aortic dissection type A has potential to facilitate stable outcomes for low-volume early-career surgeons

**DOI:** 10.3389/fsurg.2022.917686

**Published:** 2022-09-16

**Authors:** Masahiro Tsutsui, Kouhei Ishidou, Masahiko Narita, Ryohei Usioda, Yuta Kikuchi, Tomonori Shirasaka, Natsuya Ishikawa, Hiroyuki Kamiya

**Affiliations:** Department of Cardiac Surgery, Asahikawa Medical University, Asahikawa, Japan

**Keywords:** turn-up technique, anastomosis, acute aortic dissection, outcomes, surgeons

## Abstract

**Objective:**

Secure proximal anastomosis is an essential part of surgical treatment for acute aortic dissection type A (AADA). This study aimed to investigate the effectiveness of the modified turn-up technique for proximal anastomosis in AADA and compare this technique with other techniques.

**Methods:**

We divided 57 patients who underwent ascending aorta replacement for AADA into the modified turn-up technique group (group A: 36 patients) and the other technique group (group B: 21 patients). Intraoperative and postoperative course data were compared between groups A and B. In group A, we also compared early-career surgeons (practicing for <10 years after graduation) and aged surgeons (practicing for ≥10 years after graduation).

**Results:**

Preoperative patient characteristics did not differ between groups. There was a tendency toward shorter operation time in group A than in group B without statistical significance (*p* = 0.12), and the length of intensive care unit stay was significantly shorter (*p* < 0.01); the occurrence of cerebral infarction was lower (*p* < 0.01) in group A than in group B, whereas mortality and major complications other than the cerebral infarction rate did not differ between the groups. In group A, 13 patients were operated on by early-career surgeons, while 23 patients were operated on by surgeons with more than 10 years of experience. Aortic clamp time and circulatory arrest time were significantly longer in patients operated on by early-career surgeons, but outcomes were comparable.

**Conclusions:**

The modified turn-up technique was comparable to other techniques. Even for less skilled surgeons (e.g., early-career surgeons), the use of this technique may lead to stable outcomes.

## Introduction

Surgical outcomes in the treatment of acute aortic dissection type A (AADA) have improved over the years (1, 2). In AADA, proximal anastomosis is considered to be an important procedure that not only reduces the amount of bleeding during the operation (OP) but also has a positive effect on the operation time and long-term outcome. However, at present, there is still no one-of-a-kind method for AADA, and the search for the best method continues. The turn-up technique initially reported by Tamura et al. is an excellent anastomosis method with minimal bleeding from the anastomotic site (3). At our institution, several methods of proximal anastomosis for AADA were adopted, but since 2017, the modified turn-up technique has been mainly used to ensure reproducibility for the education of young surgeons. This study aimed to examine the efficacy and validity of this modified turn-up technique and compare the outcomes of this technique with those of other proximal anastomosis techniques.

## Subjects and methods

This study was done retrospectively and was conducted independently at the Asahikawa Medical University.

### Patients

Among 113 patients who underwent AADA from April 2014 to September 2020, 11 patients who underwent aortic root replacement, five patients with redo cases, and two patients who could not be followed up were excluded. In addition, the surgical procedure was limited to ascending aorta replacement using hypothermic circulatory arrest (CA) to make the conditions as similar as possible, and 27 patients underwent total arch replacement, 2 patients underwent partial arch replacement with reconstruction of at least one arch vessel, and 9 patients underwent concomitant surgery were excluded. Thus, the study included a total of 57 patients who were divided into the modified turn-up technique group (group A, 36 patients) and the other technique group (group B, 21 patients: 13 cases of the sandwich technique, 5 cases of the telescope technique, and 3 cases of the continuous suturing technique). Most of the grafts used in the surgery were J-Graft (Japan Lifeline Co, Tokyo, Japan), but only four patients used Gelweave (TERUMO Co, Tokyo, Japan). Preoperative patient characteristics, intraoperative data, and postoperative courses were compared between groups A and B. In addition, in group A, we compared aged surgeons (experience of ≥10years after graduation) with early-career surgeons (experience of <10 years after graduation). In Japan, cardiovascular surgeons usually qualify as a specialist in cardiovascular surgery around 10 years after graduation. Therefore, we set the cutoff value at 10 years.

### Surgical techniques

Cardiopulmonary bypass (CPB) was established by superior vena cava and inferior vena cava cannulation for venous return, and cannulation for the arterial return site (ascending aorta, axial artery, or femoral artery) was selected for each patient. Open distal anastomosis was performed at 25–28 °C with antegrade selective cerebral perfusion. Cross-clamping was performed in cases wherein the risk of complications with aorta cross-clamping was judged to be low, and proximal anastomosis was performed first in cases with the modified turn-up technique. In group A, retrograde cardioplegia with coronary sinus and antegrade cardioplegia through an anastomosed graft were used, and selective antegrade cardioplegia was not applied to avoid injury to the coronary ostium.

**Figure 1:**
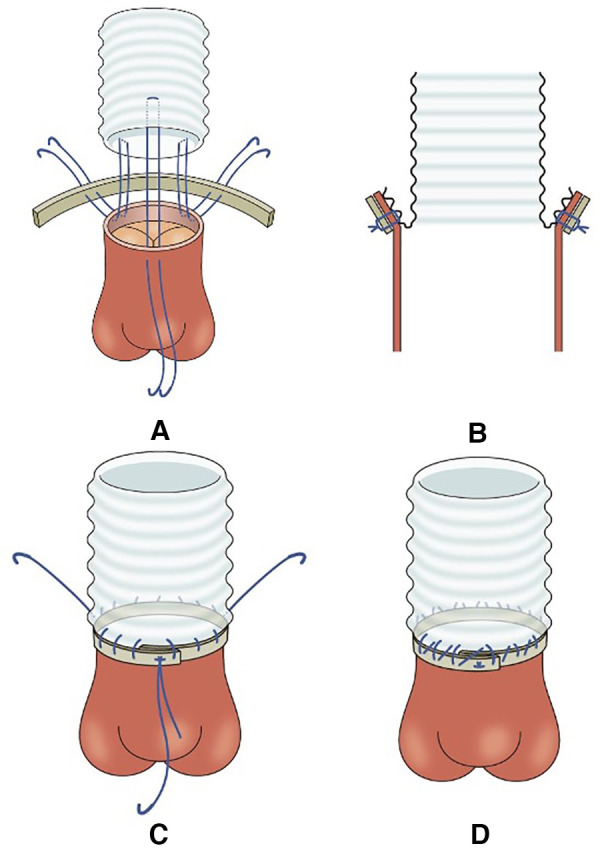
Illustration of the modified turn-up technique. (**A**) A felt strip was placed on the external border of the aortic wall, and three U-stay sutures were placed between the graft and aorta at regular intervals. (**B**) The three U-stay sutures were tied down and fixed by everting the end of the graft. (**C**) The first round of continuous sutures. The anastomosis was sutured continuously and sutures were tied each time it reached each fixation site. (**D**) The second round of continuous sutures. Sutures were tied each time it reached the fixation site same as the first.

In group A, a felt strip was placed on the external border of the aortic wall, three U-stay sutures were placed between the graft and aorta at regular intervals, and the U-stay sutures were tied down and fixed by everting the end of the graft. Subsequently, two rounds were sutured continuously and tied each time they reached each fixation site (Figure 1). When using this technique, it was necessary to use different grafts for proximal and distal anastomosis and then anastomose the grafts to each other.

In the sandwich technique, a felt strip was placed on the internal and external borders of the aortic wall, and suturing was performed continuously by everting the end of the graft. In the telescope technique, a felt strip was placed on the external border of the aortic wall, and suturing was performed by inversing the end of the graft. In the continuous suturing technique, a felt strip was placed on the external border of the aortic wall, and suturing was performed continuously by everting the end of the graft.

### Statistical analysis

Statistical analysis was conducted using EZR (Saitama Medical Center, Jichi Medical University, Saitama, Japan) (4). Continuous variables exhibiting a normal distribution were tested using the *t*-test and continuous variables exhibiting a non-normal distribution were tested using the Mann–Whitney *U*-test. For categorical variables, Fisher's test was used. The log-rank test was used to determine the survival rate. Statistical significance was set at *p *< 0.05.

### Ethical standards

The study was approved by the Institutional Review Board of Asahikawa Medical University. Approval Number: 19207.

## Results

### Patient demographics

Table 1 shows the demographic characteristics of the patients. The average age of patients in group A was 75.3 years, and there were 16 males (44%). The average age of patients in group B was 74.2 years, and there were eight males (38%). There were no major differences in the medical history or preoperative state between groups A and B.

**Table 1:** Patient characteristics.

Characteristic	Group A	Group B	*p-*value
	*n* = 36	*n* = 21	
Age, y, average	75.3 ± 11.1	74.2 ± 12.2	0.74
Male (*n*)	16 (44.4%)	8 (38.0%)	0.78
BMI, average	23.6 ± 3.7	22.8 ± 3.2	0.43
Medical history			
HT (*n*)	28 (77.7%)	14 (66.6%)	0.37
DM (*n*)	4 (11.1%)	1 (4.7%)	0.64
HLp (*n*)	9 (25.0%)	5 (23.8%)	1.00
HD (*n*)	1 (2.7%)	1 (4.7%)	1.00
PCI history (*n*)	1 (2.7%)	2 (9.5%)	0.54
Preoperative state			
Preoperative CPR (*n*)	2 (5.5%)	0 (0%)	0.52
Preoperative shock (*n*)	10 (27.7%)	3 (14.2%)	0.33
Malperfusion (*n*)	3 (8.3%)	3 (14.2%)	0.66

BMI, body mass index; HT, hypertension; DM, diabetes mellitus; HLp, hyperlipidemia; HD, hemodialysis; PCI, percutaneous coronary intervention; CPR, cardiopulmonary resuscitation.

### Operative outcomes and postoperative course

Table 2 shows the operative and postoperative course data. Surgery was performed by early-career surgeons for 13 patients in group A and 1 patient in group B, and there was a statistical significance between group A and group B. Group A contained one more anastomosis site; nonetheless, there were no significant differences in the operation data, including OP time, CPB time, CA time, and bleeding volume, between groups A and B. In the postoperative course, the length of stay in the intensive care unit was significantly shorter in group A than in group B. Major complications included cerebral infarction with sequelae, paraplegia, severe infections (such as mediastinitis and sepsis), postoperative myocardial infarction, and renal failure with new maintenance dialysis. There was a significant difference in the occurrence of cerebral infarction, and no significant differences were observed in other complications between groups A and B.

**Table 2:** Intraoperative and postoperative data.

Data	Group A	Group B	*p*-value
	*n* = 36	*n* = 21	
Intraoperative data			
OP time, min	296.9 ± 55.8	322.4 ± 67.2	0.12
CPB time, min	150.3 ± 26.9	146.3 ± 30.1	0.60
ACC time, min	98.6 ± 21.2	90.0 ± 25.5	0.17
CA time, min	36.1 ± 10.8	35.2 ± 11.4	0.76
Minimum temperature, °C	26.7 ± 1.7	26.6 ± 1.6	0.85
Bleeding volume, ml, median (IQR)	3,433 (1,854ー5,012)	3,271 (2,329ー4,218)	0.85
RBC infusion, unit	20.5 ± 7.5	21.3 ± 10.1	0.72
FFP infusion, unit	25.8 ± 11.5	23.8 ± 10.1	0.51
PC infusion, unit, median (IQR)	40 (27.5ー40)	40 (40ー55)	0.12
Early-career surgeon performed	13 (36.1%)	1 (4.7%)	<0.01
Postoperative data			
ICU stay, day, median (IQR)	3.7 (2–6)	7 (5–8)	<0.01
Hemostasis surgery due to postoperative bleeding	1 (2.7%)	3 (14.2%)	0.13
Prolonged ventilation	4 (11.1%)	7 (33.3%)	0.07
Major complication	5 (13.8%)	7 (33.3%)	0.10
Cerebral infarction	0 (0%)	4 (19.0%)	0.02
Paraplegia	0 (0%)	1 (4.7%)	0.36
Severe infection	4 (11.1%)	2 (9.5%)	1.00
Postoperative myocardial infarction	0 (0%)	0 (0%)	N/A
Renal failure	1 (2.7%)	0 (0%)	1.00
Reintervention	4 (11.1%)	3 (14.2%)	0.70
30-day mortality	3 (8.3%)	1 (4.7%)	1.00

OP time, operation time; CPB time, cardiopulmonary bypass time; ACC time, aortic cross clamp time; CA time, circulatory arrest time; IQR, interquartile range; RBC, red blood cell; FFP, fresh frozen plasma; PC, platelet concentrate; ICU, intensive care unit.

**Figure 2:**
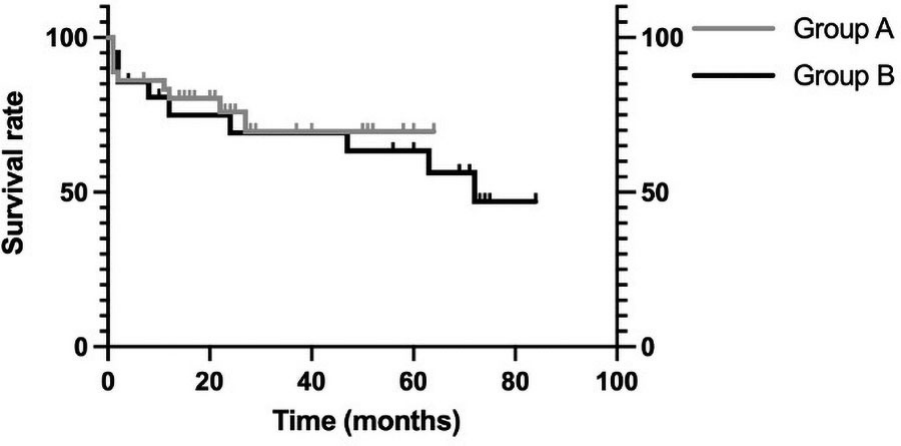
Comparison of group A and group B survival rates. There is no significant difference in the 5-year survival rate between group A and group B (69.6% vs. 63.4% *p* = 0.67).

Perioperative death occurred in three patients in group A and in one patient in group B. In the follow-up period, reintervention due to cardiovascular disease occurred in four patients in Group A and three patients in Group B: two aortic root replacements for pseudo-aneurysm at the proximal anastomosis site and vascular graft infection, one thoracic endovascular aortic repair for acute aortic dissection type B, and one total arch replacement for aortic aneurysm dissection at the distal arch in Group A; one aortic root replacement for residual dissection at the aortic root, one transcatheter aortic valve implantation for aortic valve stenosis, and one multivalve surgery for heart failure due to valvular disease in Group B. There was no significant difference between the two groups in this regard. Other postoperative data showed no significant differences. The 5-year survival rates were 69.6% in Group A and 63.4% in Group B, with no significant difference between the two groups (Figure 2).

Table 3 shows the comparison within Group A; aortic cross clamp (ACC) time and CA time were significantly shorter for aged surgeons, but there were no significant differences in other operation data. Furthermore, there was no significant difference in any of the postoperative data.

**Table 3:** Comparison of early-career surgeons and aged surgeons within group A.

Data	Early-career surgeons group	Aged surgeons group	*p-*value
	*n *= 13 (%)	*n *= 23 (%)	
Intraoperative data			
OP time, min	305.0 ± 35.8	292.3 ± 64.8	0.55
CPB time, min	156.8 ± 16.5	146.7 ± 31.0	0.28
ACC time, min	109.6 ± 15.1	92.3 ± 21.9	0.01
CA time, min	45.0 ± 6.7	31.1 ± 9.4	<0.01
Bleeding volume, ml, median (IQR)	3,362 (2,267ー4,705)	3,459 (1,843ー6,631)	0.69
Postoperative data			
ICU stay, day, median (IQR)	5 (3–6)	6 (4.5–7)	0.28
Hemostasis surgery due to postoperative bleeding	0 (0%)	1 (4.7%)	1.00
Prolong ventilation	0 (0%)	4 (19.0%)	0.27
Major complication	0 (0%)	5 (21.7%)	0.13
Cerebral infarction	0 (0%)	0 (0%)	N/A
Paraplegia	0 (0%)	0 (0%)	N/A
Severe infection	0 (0%)	4 (19.0%)	0.27
Postoperative myocardial infarction	0 (0%)	0 (0%)	N/A
Renal failure	0 (0%)	1 (4.7%)	1.00
Reintervention	2 (5.5%)	2 (9.5%)	0.60
30-day mortality	0 (0%)	3 (14.2%)	0.28

OP time, operation time; CPB time, cardiopulmonary bypass time; ACC time, aortic cross clamp time; CA time, circulatory arrest time; IQR, interquartile range; ICU, intensive care unit.

## Discussion

The crucial findings of the present study are as follows: (1) although 36% of the modified turn-up technique was performed by early-career surgeons, this technique brought comparable outcomes to other anastomosis techniques and there was no influence of surgeons' experience on outcome in patients who underwent the modified turn-up technique. (2) Therefore, this technique might be useful to facilitate surgical training in low-volume centers.

### Modified turn-up technique has the potential to be comparable with other anastomosis techniques regarding safety

Proximal anastomosis is a very important part of the operation of acute aortic dissection type A. There are various anastomosis methods, and reports of anastomosis and anastomosis reinforce methods are still ongoing (3, 5–8). After some transitions, the modified turn-up technique has been the choice for proximal anastomosis if root replacement could be avoided at our institution. In our opinion, this technique has some advantages over other techniques because of the following reasons: (1) proximal anastomosis can be performed in a very good surgical field without being bothered by an aortic clamp, (2) hemostasis can be checked immediately after finishing anastomosis by administrating a cardioplegic solution through the vascular graft, (3) therefore, an additional stitch is very easy because it can be done without blood pressure and (4) cannulation into the coronary ostia can be avoided and, therefore, the risk of coronary injury seems to be very low.

Among various strong points, the most important one is reproducibility. In this study, ACC time and CA time of aged surgeons were significantly shorter than those of early-career surgeons, whereas OP time and CPB time were not significantly affected by the experience of surgeons. Moreover, there was no difference in postoperative course, and there was even a trend showing a better course for early-career surgeons. This result suggested that the modified turn-up technique would have reproducibility independent of surgeons' experience in surgical treatment for AADA.

On the other hand, this technique has an obvious disadvantage, i.e. one additional anastomosis between two grafts is required, which might result in prolonged operation time and increased bleeding amount. However, in this study, bleeding volume and intraoperative time factors showed no significant difference between the modified turn-up technique and others. Therefore, we consider that the disadvantage may be subclinical and neglectable.

### Modified turn-up technique seems to have some merit for low-volume surgeons in low-volume centers

The influence of surgeon and institutional procedure volume on the operative result is reported by a lot of research. Chikwe et al. reported that patients undergoing emergency repair of acute aortic dissection by lower-volume surgeons and centers have approximately double the risk-adjusted mortality of patients undergoing repair by the highest volume care providers (9). Bashir et al. reported that surgeons with a mean annual volume of AADA procedure <4 had significantly higher in-hospital mortality rates in comparison with surgeons with a mean annual volume AADA procedure ≥4 (10). However, centralization of cardiac surgery and surgical cases of AADA is very difficult because of political and geographical reasons in Japan. In Japan, there are approximately 600 institutions performing cardiac surgery and the annual surgical cases of AADA are approximately 6,000 (11); thus, the surgical volume of AADA per institute is annually only 10 cases on average. Moreover, in geographically large but low population density areas such as north Hokkaido where our institute is, centralization would be impossible; otherwise, patients suffering from AADA must tolerate very long distance transfers over 300 km. In the present study, the annual institutional volume was approximately 20 cases and all of the early-career surgeons had experience as the first operator for AADA in less than 10 cases, but this factor of surgeons' experience did not impair the surgical outcome. This finding might suggest that the disadvantage of a low-volume center/surgeon could be overcome by a reproducible operative method, i.e., the modified turn-up technique.

### Study limitations

This study has some limitations. First, this is a retrospective, single-institutional, and low-volume study. Second, heterogeneity of surgeons' experiences and skills not only between aged and early-career surgeons, but also among aged surgeons could influence the outcome. Third, selection bias of operating surgeons was unavoidable and sicker patients would have been operated on by aged surgeons. Fourth, techniques other than proximal anastomosis were not completely uniform. This could have affected the operational data.

## Conclusion

In the present study, despite of aggressive participation of early-career surgeons doing the modified turn-up technique, the outcome of this technique was comparable to other anastomosis techniques mainly done by aged surgeons. Moreover, in patients who underwent the modified turn-up technique, there was no influence of surgeons' experiences on the outcome. Therefore, this technique appeared to be feasible for early-career and/or low-volume center surgeons.

## Data Availability

The original contributions presented in the study are included in the article/Supplementary Material, further inquiries can be directed to the corresponding author.
